# Vemurafenib induces senescence in acute myeloid leukemia and myelodysplastic syndrome by activating the HIPPO signaling pathway: implications for potential targeted therapy

**DOI:** 10.1186/s13062-023-00451-0

**Published:** 2024-01-04

**Authors:** Qiao Zhou, Jiamin Zhang, Jingsong Zhang, Simin Liang, Duo Cai, Han Xiao, Yu Zhu, Wenqiong Xiang, Fernando Rodrigues-Lima, Jianxiang Chi, Fabien Guidez, Li Wang

**Affiliations:** 1 Department of Hematology, The First Affiliated Hospital of Chongqing Medical University, No.1 Youyi Road, Yuzhong District, Chongqing, 400000 People’s Republic of China; 2 Unité de Biologie Fonctionnelle et Adaptative, Université Paris Cité, CNRS UMR 8251, Paris, France; 3 Center for the Study of Hematological Malignancies, Karaiskakio Foundation, Nicosia, Cyprus; 4 UMR1231 Inserm/uB/AgroSup, Université de Bourgogne, 7 boulevard Jeanne d’Arc 21079 DIJON Cedex, DIJON, France

**Keywords:** *BRAF*, Vemurafenib, Senescence, HIPPO signaling pathway, MDS, AML

## Abstract

**Background:**

The outcome of Acute myeloid leukemia (AML) and myelodysplastic syndrome (MDS) remain dismal despite the development of treatment. Targeted therapy is gaining more and more attention in improving prognosis.

**Methods:**

Expression of *BRAF* was analyzed by RT-qPCR in AML and MDS patients. Cells viability treated by drugs was measured by CCK-8 assay. Network pharmacology and RNA-sequence were used to analyze the mechanism of drugs and verified in vitro and xenograft tumor model.

**Results:**

Here we showed that *BRAF* was overexpressed in AML and MDS patients, and correlated with poor prognosis. The *BRAF* inhibitor-Vemurafenib (VEM) could significantly induce senescence, proliferation inhibition and apoptosis in AML cells, which can be enhanced by Bortezomib (BOR). This inhibitory effect was also verified in CD34 + cells derived from AML patients. Mechanistically, we showed that VEM combined with BOR could turn on HIPPO signaling pathway, thereby inducing cellular senescence in AML cells and xenograft mouse.

**Conclusions:**

Taken together, our findings demonstrate a significant upregulation of *BRAF* expression in AML and MDS patients, which is associated with unfavorable clinical outcomes. We also discovered that the *BRAF* inhibitor Vemurafenib induces cellular senescence through activation of the HIPPO signaling pathway. Analysis of *BRAF* expression holds promise as a prognostic indicator and potential therapeutic target for individuals with AML and MDS.

**Supplementary Information:**

The online version contains supplementary material available at 10.1186/s13062-023-00451-0.

## Background

Acute myeloid leukemia (AML) and myelodysplastic syndrome (MDS) which is considered the preleukemia status, are among the most common hematological malignancies in adults [1–3]. Although the majority of AML and MDS enter remission upon standard therapy, poor prognosis is considerable due to primary refractoriness, relapse, or treatment-related mortality. In this scenario, targeted therapy has gradually become a hot field in the treatment of hematological malignancies Because of good efficacy and few side effects.

The *BRAF* gene is located upstream of MAPK pathway and encodes RAF family serine/threonine protein kinases. Abnormal *BRAF* gene expression can cause continuous activation of MAPK pathway, and lead to tumorigenesis [4]. *BRAF* could bind to MST2 [5], an important upstream molecule of HIPPO signaling pathway, then regulates the phosphorylation of YAP and TAZ, allowing non-phosphorylated YAP/TAZ enter the nucleus to activate TEAD and turn off the HIPPO signaling pathway [6, 7], which is involved in cell proliferation, apoptosis, and senescence [8]. Previous studies suggested that *BRAF* gene mutations were associated with the development of treatment-associated AML (t-AML) [9, 10]. Our data showed that *BRAF* was overexpressed in MDS and AML patients and correlated with poor clinical outcomes. Notably, Vemurafenib (VEM), a first-generation *BRAF* inhibitor that targets *BRAF*^V600E^ mutation [11, 12] can markedly induce senescence and apoptosis in AML cells. Yet the role of *BRAF* in AML and the underlying mechanism by which VEM inhibits AML cells has not been fully elucidated. Therefore, we sought to investigate the therapeutic potential of VEM and its underlying mechanism in AML and MDS aiming to propose a new alternative targeted therapy in the treatment.

## Methods

### Patients and clinical characteristics

Bone marrow samples were obtained from 101 consecutive newly diagnosed AML patients, 54 MDS patients and 18 healthy controls at the Hematology Department of the First Affiliated Hospital of Chongqing Medical University from 2014 to 2022. The diagnosis of AML and MDS were according to the 2016 World Health Organization classification of myeloid neoplasms and acute leukemia [13]. Details of the treatment regimens was according to 2017 European LeulemieNet (ELN) management of AML and Chinese guidelines for treatment of MDS (2019) [14, 15]. In the meantime, 21 AML patients received hematopoietic stem cell transplantation (HSCT). We gathered clinical data form patients’ electronic medical records system. Overall survival (OS) was defined as the period from diagnosis to the endpoint, such as death or the last time of follow-up time (August 31th, 2022). Bone marrow mononuclear cells (BMMCs) were separated from the participants using Lymphocyte Separation Medium (TBD, China, LTS1077) using density-gradient centrifugation. This study was approved by the Ethics Committee of the First Affiliated Hospital of Chongqing Medical University (2021-684).

### Cell lines

The human AML cell line SKM-1 was provided by Professor Zhou Jianfeng at Tongji Medical College, Huazhong University of Science Technology and cultured in a RPMI-1640 medium prior to its supplementation with 10% fetal bovine serum (Gibco-BRL, Grand Island, NY, USA) in a humid atmosphere at 37 °C with 5% CO_2_. SKM-1 cells were constructed from a 70-year-old male patient with acute monocytic leukemia (AML-M5). The human AML cell line MOLM-13 was provided by Professor Tong Hongyan at the First Affiliated Hospital of Zhejiang University and cultured in a cytiva-IMDM medium prior to its supplementation with 10% fetal bovine serum (cytiva, Hyclone Laboratories, Utah, USA) in a humid atmosphere at 37 °C with 5% CO2. MOLM-13 is an immortalized AML cell line derived from AML cells from a relapsed patient. To prevent mycoplasma contamination, we regularly used Mycoplasma Off™ (a German reagent designed for mycoplasma elimination) within the cell incubator on a weekly basis.

### Immunofluorescence (IF)

SKM-1cells were treated with VEM (20 µM), or Bortezomib (BOR, 10 nM), for 48 h. Meanwhile, VEM, at a concentration of 5 µM, or BOR, at a concentration of 4 nM, was added to 1 million MOLM-13 cells for 48 h. AML cell lines were collected and washed three times with phosphate buffer saline (PBS), then blocked in 4% paraformaldehyde, and 0.1% Triton X-100 at room temperature for 1 h. Then AML cell lines were incubated with primary antibody overnight at 4 °C. Primary antibodies were used: TRF2 (Rabbit, Abcam-ab108997), Lamin B1 (rabbit, Abcam-ab133741). AML cell lines were then washed three times in PBS and incubated with secondary antibodies for 1 h at room temperature. The following secondary antibodies were applied in our study: FITC Goat Anti-Rabbit IgG (AS011). Finally, AML cell lines were washed three times (each time for 5 min) with PBS and then incubated with CrystalMount with DAPI for 10 min. Fluorescent images were captured by using a Leica confocal microscope (Leica TCS SP8 SR).

### Immunohistochemistry (IHC)

Tumors were blocked in 4% paraformaldehyde. Next, the slides were incubated with primary antibodies against YAP1 (Rabbit, Abclonal-A1002), and TAZ (Rabbit, Abclonal-A8202) at 4 °C, followed by incubation with horseradish peroxidase (HRP)-conjugated secondary antibody for 30 min and then staining with diaminobenzidine. The images were captured and evaluated using Image-Pro 6.0 software (Media Cybernetics, USA).

### RNA-seq and data analysis

Total RNA was isolated from SKM-1 cells treated with BOR/ VEM, and a combination of both agents using the RNeasy Mini Kit (Qiagen). The mRNA library was constructed using the Smart-Seq V4 Ultra Low Input RNA Kit for sequencing (Takara) according to the manufacturer’s instruction. Libraries were sequenced by the Illumina HiSeq 2000 platform as 150-bp pair-ended reads. Reads were aligned using bowtie v0.12.9. Fragments per kilobase per million (FPKM) estimation was performed with Cufflinks v2.1.1, aligned reads were counted with HTSeq (a Python framework to work with high-throughput sequencing data). Differential expression analysis of two conditions/groups (two biological replicates per condition) was performed with DEseq2, and prior to differential expression analysis without biological replicates, for each sequenced library, the read counts were adjusted by edgeR program package through one scaling normalized factor. Differentially expressed genes were selected using a cutoff *P* value of < 0.01 (false discovery rate adjusted for multiple testing).

### Network pharmacological analysis

Drug bank, SEA Search Server, Therapeutic Target Database, TargetNet, CANSAR, SwissTargetPrediction1 and PharmMapper2 were used to establish the targets of VEM and BOR. Genomic targets of MDS, AML and AML-MDS were obtained from DisGeNet, GeneCards3 and OMIM, and overlapping genes were collected. Subsequently, VEM- and BOR-associated targets were mapped to these overlapping disease-targets, followed by therapeutic targets of VEM and BOR against AML-MDS were obtained. The STRING database4 was used to obtain interactions among potential targets of VEM, BOR and the diseases. Protein interactions with a combined score > 0.4 were selected. As a result, 1933 genes were collected using DisGeNET, OMIM and GeneCards database to research AML-related targets without duplicated genes. Furthermore, 390 and 275 predicted targets of BOR and VEM were screened from the PharmMapper, SEA, SwissTargetPredict, TargetNet database, canSAR BLACK, Therapeutic Target and Drug bank database. Cytoscape 3.7.1 was used to construct and analyze the protein–protein interaction (PPI) network. DAVID database5 was used to perform Gene Ontology (GO) and Kyoto Encyclopedia of Genes and Genomes (KEGG) pathway enrichment analyses.

### Xenograft tumor model

A total of 1*10^7^ MOLM-13 cells was injected subcutaneously in the right lower dorsal side of mice. The experimental groups consisted of 6-week-old mice housed in a pathogen-free facility. While the tumors were measurable (100–150 mm^3^) after two weeks, mice were randomly divided into four groups (4 mice for each group): Control group (equal volume of vehicle), BOR group, VEM group, and combined group. We treat mice with BOR (1 mg/kg; n = 4), and VEM (20 mg/kg, n = 4), or the combination (1 mg/kg + 5 mg/kg; n = 4) for 3 weeks. We measured mice's weight and tumor size every two days using vernier calipers. The volume of tumor was calculated by the following formula: V = 0.5*a*b^2^, where a and b were the length and width of the tumors respectively. All the animal studies performed were approved by the Animal Ethics Committee of Chongqing Medical University (2021-601).

### Statistical analysis

All data was presented as means ± standard deviation (SD) and statistical analyses were performed using GraphPad Prism 8 (GraphPad Software Inc., San Diego, CA, United States). The Kaplan–Meier (KM) method and COX proportional hazards regression model was performed to identify the prognostic value of clinical features and gene mutations. The results were analyzed using one-way and two-way ANOVA followed by the Bonferroni post hoc test. Furthermore, for co-mutated genes analysis, Kaplan–Meier survival analysis, nomogram analysis and the landscape of somatic mutations in patients was performed by HIPLOT online website (https://hiplot.com.cn). *P *< 0.05 represents statistical significance. All experiments were performed in triplicates.

## Results

### BRAF was overexpressed in AML patients and correlated with poor prognosis

**Fig. 1:**
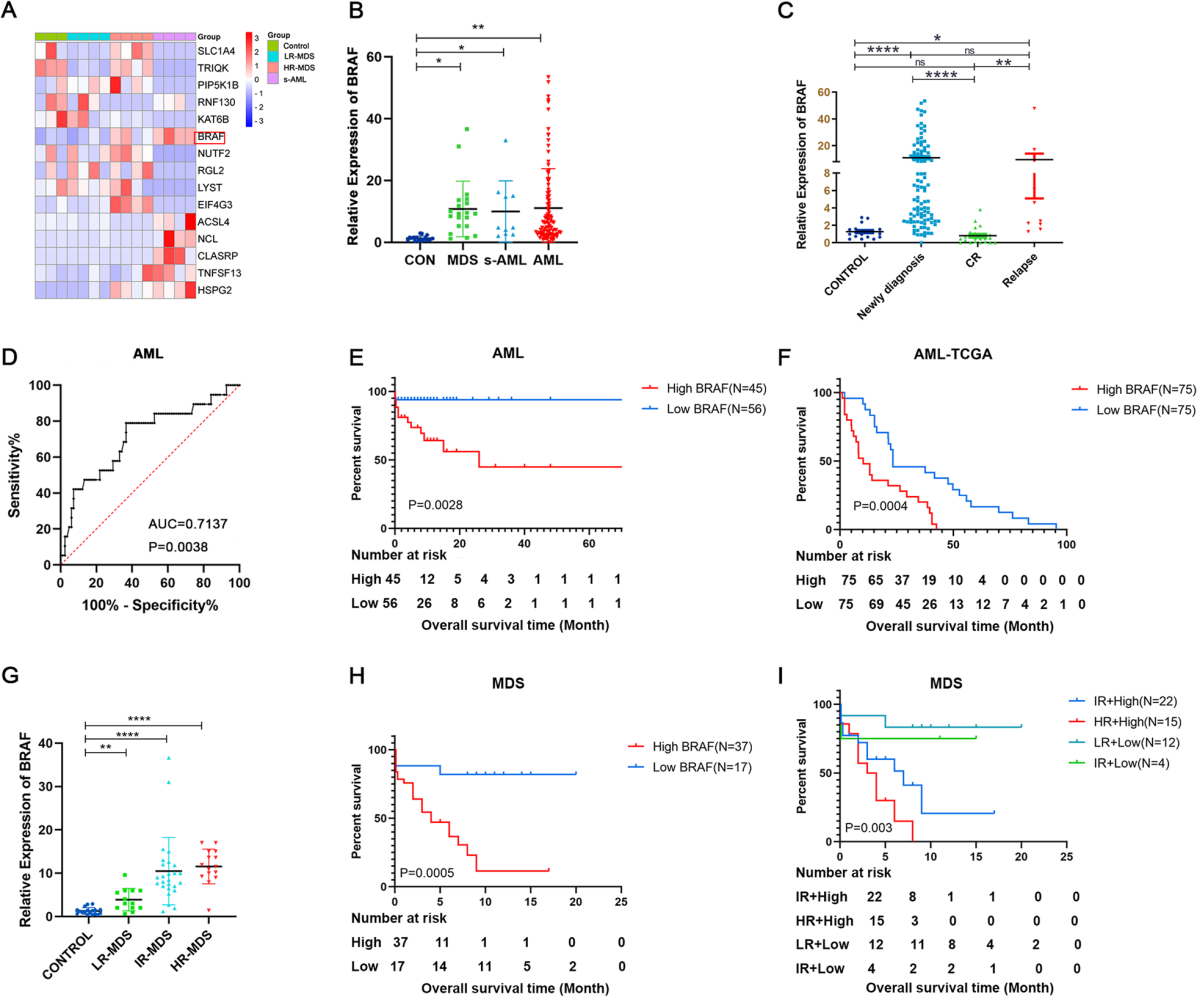
High expression of BRAF indicates poor prognosis in AML and MDS patients. **A** Hierarchical clustering of differentially expressed genes in MDS and AML patients compared to healthy controls; **B** Relative expression level of BRAF was normalized to β-actin in healthy controls, MDS patients, AML patients and sAML patients; **C** Relative expression level of BRAF was normalized to β-actin in different stage of AML by RT-qPCR; **D** ROC curve analysis of BRAF expression for discriminating AML patients from controls; **E** Prognostic impact of BRAF expression on AML patients; **F** Prognostic impact of BRAF expression on AML patients in TCGA database; **G** Relative expression level of BRAF was normalized to β-actin in healthy controls, LR-MDS patients, IR-MDS patients, and HR-MDS patients; **H** Prognostic impact of BRAF expression on MDS patients, **I** Survival-Analysis of MDS patients with different BRAF experssion and IPSS group. *P < .05; **P < .01; ***P < .001; ****P < .001. Error bars indicate the SD

To determine the role of *BRAF* in the pathogenesis of AML, we first explored the *BRAF* expression in our RNA-seq analysis including bone marrow sample from patients with MDS, AML and secondary AML (sAML) which arises from previous MDS. The *BRAF* expression was higher in high risk MDS (HR-MDS) and low risk MDS (LR-MDS) compared with healthy control (Fig. 1A) and the highest expression was observed in sAML patients. Based on these findings, the transcript level of *BRAF* in bone marrow samples was assessed in 101 adults with AML patients including 10 sAML, 54 MDS patients and 28 healthy controls. *BRAF* expression was significantly higher in MDS, sAML, and AML patients when compared with controls (Fig. 1B). We then compared *BRAF* transcript levels in subjects with AML, in complete remission (CR) and relapse. Transcript levels decreased to a level equivalent to that in normal subjects in CR but increased at relapse (Fig. 1C).

The diagnostic value of *BRAF* in AML patients was analyzed using ROC curves with a AUC value of 0.7137, sensitivity of 78.95% and specificity of 63.41% (Fig. 1D).

To explore the prognostic impact of *BRAF*, AML patients with prognostic information were divided into 2 cohorts with high or low *BRAF* levels. Characteristic were summarized in Table 1. Overall survival time (OS) in patients with high *BRAF* expression was significantly shorter than those with low *BRAF* expression (Fig. 1E). To verify this conclusion, the correlation of *BRAF* expression level and survival were analyzed in AML patients from online database. Data from ONCLOC also showed that high expression of *BRAF* was significantly associated with a shorter overall survival (Fig. 1F).

**Table 1:** Clinical characteristics of AML patients

Patients Parameters	All Patients (n = 101)	*BRAF* expression		*P*
		High (n = 45)	Low (n = 56)	
Sex, male/female	49/52	20/25	29/27	0.5344
Median age, year, (range)	48.71 (15–80)	52.58 (20–80)	45.63 (15–79)	0.0377
Median WBCx109/L, (range)	45.07 (0.82–496.28)	50.84 (0.95–396.28)	40.46 (0.82–496.28)	0.5412
Median HGB, g/L, (range)	81.02 (25–159)	80.25 (41–136)	81.64 (25–159)	0.7899
Median PLTx109/L, (range)	70.10 (2–866)	68.61 (2–479)	71.31 (6–866)	0.9036
*FAB classification*				
M1	3 (2.97%)	2 (4.44%)	1 (1.79%)	
M2	28 (27.72%)	11 (24.44%)	17 (30.36%)	
M3	6 (5.94%)	4 (8.89%)	2 (3.57%)	
M4	13 (12.87%)	8 (17.78%)	5 (8.93%)	
M5	26 (25.74%)	8 (17.78%)	18 (32.14%)	
M6	/	/	/	
M7	/	/	/	
Others	15 (14.85%)	8 (17.78%)	7 (12.50%)	
sAML	10 (9.90%)	4 (8.89%)	6 (10.71%)	
*Karyotypes*				
Abnormal karyotypes	21 (20.79%)	11 (24.44%)	10 (17.86%)	0.5658
*Gene mutation*				
WT1	29 (28.71%)	13 (28.89%)	16 (28.57%)	0.9724
NPM1	9 (8.91%)	8 (17.78%)	1 (1.79%)	0.0047
DNMT3A	9 (8.91%)	5 (11.11%)	4 (7.14%)	0.4915
FLT3-ITD	12 (11.88%)	8 (17.78%)	4 (7.14%)	0.1026
FLT3-TKD	4 (3.96%)	4 (8.89%)	/	0.0227
FLT3	2 (1.98%)	1 (2.22%)	1 (1.79%)	0.8772
AML-ETO	5 (4.95%)	2 (4.44%)	3 (5.36%)	0.8356
TET2	5 (4.95%)	2 (4.44%)	3 (5.36%)	0.8356
C-KIT	5 (4.95%)	2 (4.44%)	3 (5.36%)	0.8356
IDH1	4 (3.96%)	3 (6.52%)	1 (1.79%)	0.2152
IDH2	3 (2.97%)	3 (6.52%)	/	0.0505
SRSF2	3 (2.97%)	1 (2.22%)	2 (3.57%)	0.6949
NRAS	3 (2.97%)	/	3 (5.36%)	0.1173
TP53	2 (1.98%)	1 (2.22%)	1 (1.79%)	0.8772
RUNX1	2 (1.98%)	/	2 (3.57%)	0.2042
ASXL1	2 (1.98%)	/	2 (3.57%)	0.2042
SF3B1	2 (1.98%)	/	2 (3.57%)	0.2042
*Risk category*				0.002
Favorable	2 (1.98%)	/	2 (3.57%)	
Intermediate	90 (89.11%)	41 (91.11%)	49 (87.50%)	
Adverse	9 (8.91%)	4 (8.89%)	5 (8.93%)	
*Treatment regimen*				0.001
Chemotherapy-only	80 (79.21%)	40 (88.89%)	40 (71.43%)	
HSCT	21 (20.79%)	5 (11.11%)	16 (28.57%)	

*WBC* White blood cell count, *PLT* Platelets count, *HGB* Hemoglobin content, *HSCT* Hematopoietic stem cell transplantation, *sAML* Secondary acute myeloid leukemia

We further analyzed the prognostic value of *BRAF* in MDS patients. transcript level of *BRAF* in patients with MDS were significantly higher when compared with that of healthy controls, and increased along with the risk stratification (Fig. 1G). Similar with AML, OS in MDS patients with high *BRAF* expression was also significantly shorter than those with low *BRAF* expression (Fig. 1H, I).

### High BRAF expression was correlated with age and gene mutation in AML

**Fig. 2:**
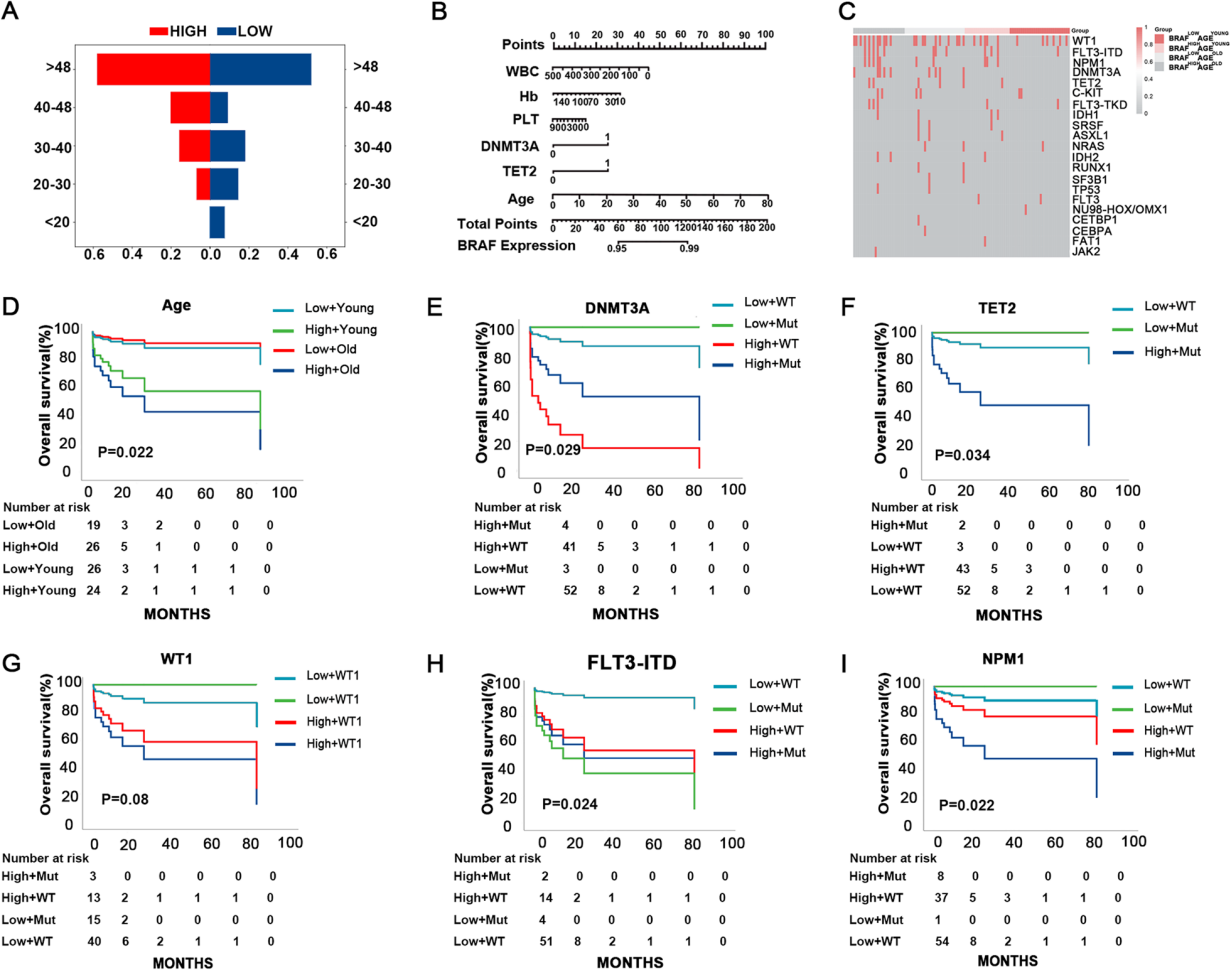
High BRAF expression was correlated with age and gene mutation in AML. **A** The distribution of age in AML patients with low and high BRAF expression; **B** A merged score for BRAF expression prediction established by nomogram based on the age, gene mutations and other clinical characters; **C** Heat map of AML patients grouped by age and BRAF expression level; **D** Survival analysis of AML patients based on age and BRAF transcript level; **E** Survival analysis of AML patients based on DNMT3A mutation and BRAF transcript level; **F** Survival analysis of AML patients based on TET2 mutation and BRAF transcript level; **G** Survival analysis of AML patients based on WT1 expression and BRAF transcript level; **H** Survival analysis of AML patients based on FLT3-ITD mutation and BRAF transcript level; **I** Survival analysis of AML patients based on NPM1 mutation and BRAF transcript level

Distribution analysis showed that patients with high *BRAF* expression tend to older than those with low *BRAF* expression (Table 1 and Fig. 2A), and the expression of *BRAF* was positively correlated with age as indicated by nomogram analysis (Fig. 2B). In addition, gene abnormalities were more frequently observed in older AML patients with high *BRAF* expression, while younger patients with low *BRAF* expression harbor the fewest mutations and co-mutated genes (Fig. 2C). Meanwhile, we separately analyzed the prognostic impact of *BRAF* according to age stratification. Data showed that even in older patients which were generally considered to have a worse outcome, *BRAF* expression level can still distinguish OS (Fig. 2D). And in younger patients, high expression level of *BRAF* can further identity a population with worse prognosis (Fig. 2D).

AML patients with older age were more likely to carry gene mutation such as TET2 and DNMT3A, and these mutations were considered to involve in accumulation of cellular damage and promote progression of MDS to AML [16]. To avoid confounding bias, OS was analyzed separately in different stratifications. Subjects with high expression level of *BRAF* were still correlated with worse OS in stratification analysis based on common prognostic factors including WT1 expression, DNMT3A, TET2, FLT3-ITD and NPM1 mutation (Fig. 2E–I). Meanwhile, data in the TCGA database also showed that high *BRAF* expression could identify AML patients with worse outcome regardless of TET2 and DNMT3A mutations (Additional file 1: Fig. S1A–C), and AML patients with both high *BRAF* expression and these mutations were more likely to distributed in older population (Additional file 1: Fig. S1D–E).

To further explore the survival curve and risk factors of AML patients, the KM method and the multivariate Cox proportional hazard regression model were used to determine the expression level of *BRAF* and the patients’ characteristic. As shown in Table 2, the expression level of *BRAF* was correlated with risk category and treatment regimen in AML (Table 2).

**Table 2:** The variables in univariate analysis and multivariate analysis of survival and their *P* values for acute myeloid leukemia

Variables	Univariate analysis HR (CI 95%)	*P* value	Multivariate analysis HR (CI 95%)	*P* value
AGE	1.032 (0.999–1.066)	0.057		
SEX	0.840 (0.324–2.178)	0.720		
WBC	1.001 (0.995–1.006)	0.821		
Hb	1.000 (0.982–1.018)	0.991		
PLT	1.001 (0.998–1.003)	0.671		
BRAF expression	1.042 (1.015–1.069)	0.002	1.027 (0.991–1.064)	0.140
AGE ≥ 48 years	0.599 (0.227–1.582)	0.301		
WT1 mutated and wt	0.770 (0.272–2.174)	0.621		
FLT3-ITD mutated and wt	2.078 (0.582–7.418)	0.260	1.176 (0.310–4.455)	0.812
NPM1 mutated and wt	0.589 (0.077–4.479)	0.609		
DNMT3A mutated and wt	1.332 (0.302–5.882)	0.705		
TET2 mutated and wt	0.045 (0.000–526.014)	0.517		
C-KIT mutated and wt	0.556 (0.072–4.278)	0.573		
FLT3-TKD mutated and wt	1.344 (0.176–10.238)	0.775		
IDH1 mutated and wt	0.047 (0.000–9361.29)	0.624		
SRSF mutated and wt	0.048 (0.00–55212.04)	0.669		
ASXL1 mutated and wt	0.048 (0.00–162969.9)	0.692		
NRAS mutated and wt	0.047 (0.000–5527.25)	0.607		
IDH2 mutated and wt	0.047 (0.00–13658.72)	0.634		
Risk category	0.488 (0.246–0.969)	0.041	0.818 (0.326–2.054)	0.669
Treatment regimen	0.294 (0.125–0.692)	0.005	0.404 (0.177–0.923)	0.032

*WBC* White blood cell count, *PLT* Platelets count, *HGB* Hemoglobin content, *HR* Hazard ratio

### BRAF inhibitor VEM and BOR synergistically inhibited proliferation and promoting apoptosis in AML cells

**Fig. 3:**
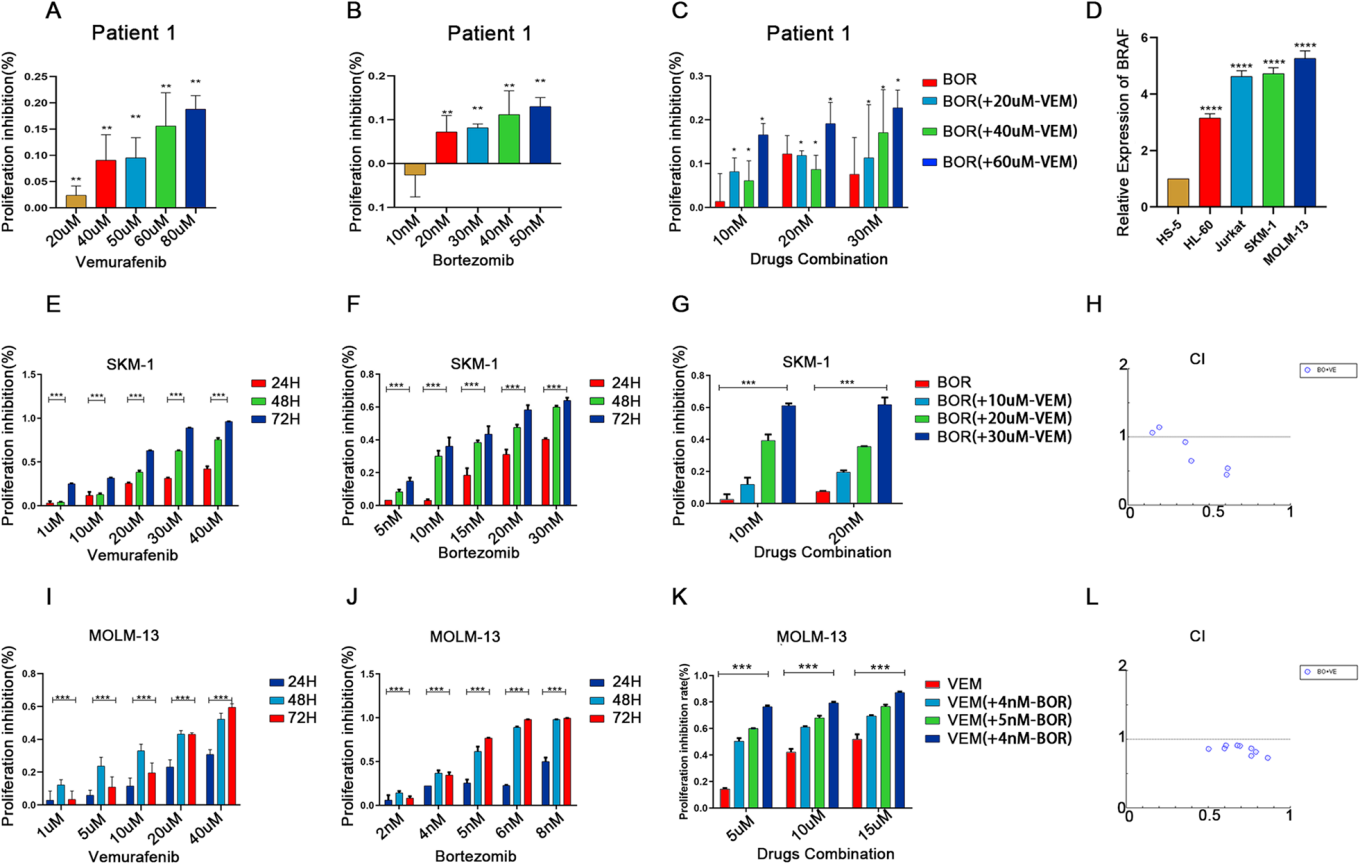
VEM and BOR inhibited the proliferation of AML cells. **A**–**C** CD34 + Cells derived from AML patient were treated with VEM or/and BOR for 48 h. Cell viability was determined by CCK-8 assay; **D** Relative expression level of BRAF was normalized to β-actin in cell lines by RT-qPCR; **E**–**F** SKM-1 cells were exposed to VEM and BOR alone, Cell viability was determined by CCK-8 assay; **G** SKM-1 cells were treated with VEM combined with BOR for 48 h; **H** Combination index values were calculated with CompuSyn software. CI < 1 indicates synergy; CI = 1 is additive; and CI > 1 means antagonism. CI, combination index; Fa, effect levels; **I**–**J** MOLM-13 cells were exposed to VEM and BOR alone; **K** MOLM-13 cells were treated with VEM combined with BOR for 48 h; **L** Combination index values were calculated with CompuSyn software. *P < .05; **P < .01; ***P < .001; ****P < .001. Data was presented as mean ± SD of three independent experiments

Since high *BRAF* expression was observed in patients with AML and MDS, and correlated with OS, we sought to test whether VEM, a *BRAF* inhibitor have therapeutic potential in AML cells. We found that VEM could inhibit the proliferation of CD34^+^ cells derived from AML patients with high *BRAF* expression in a dose dependent manner (Fig. 3A). BOR was usually used to enhanced the efficiency in many therapies in AML, we then tested whether the combination of VEM and BOR could synergistically inhibit AML cells. BOR alone can significantly inhibit cell growth (Fig. 3B). Furthermore, VEM combined BOR could synergistically inhibited the proliferation of CD34^+^ cells from AML patients (Fig. 3C).

We then tested the expression level of *BRAF* in several leukemia cell lines. SKM-1 and MOLM-13 cells derived from sAML with high *BRAF* expression were used in following experiments (Fig. 3D). CCK-8 assay showed a time and dose-dependent cell viability inhibition by VEM (Fig. 3E and I). A time and dose-dependent cell viability inhibition was also observed sAML cell lines treated with BOR (Fig. 3F and J). Then sAML cell lines were treated in a low dose of BOR combined with different concentrations of VEM for 48 h. The CCK-8 assay showed a synergistic proliferation inhibition of the combination of VEM and BOR (Fig. 3 G and K), as revealed by the combination index (CI) (Fig. 3H and L).

SKM-1 and MOLM-13 cells treated with VEM and/or BOR were then analyzed by flow cytometry for apoptosis rate. As expected, VEM combined with BOR can synergistically promote the apoptosis in both sAML cell lines compared with BOR or VEM used alone (Additional file 1: Fig. S2A–D). And the combination of these two drugs could promote the expression of BAX and the cleavage of Caspase3, while inhibiting the expression of BCL2 (Additional file 1: Fig. S2E–J).

### VEM and BOR synergistically inducing cellular senescence in AML cells

Considering *BRAF* inhibitor VEM and BOR can inhibit cell growth and promote apoptosis, and cell behavior changes usually due to the induction of cellular senescence. We sought to invested the impact of *BRAF* on cell senescence.

**Fig. 4:**
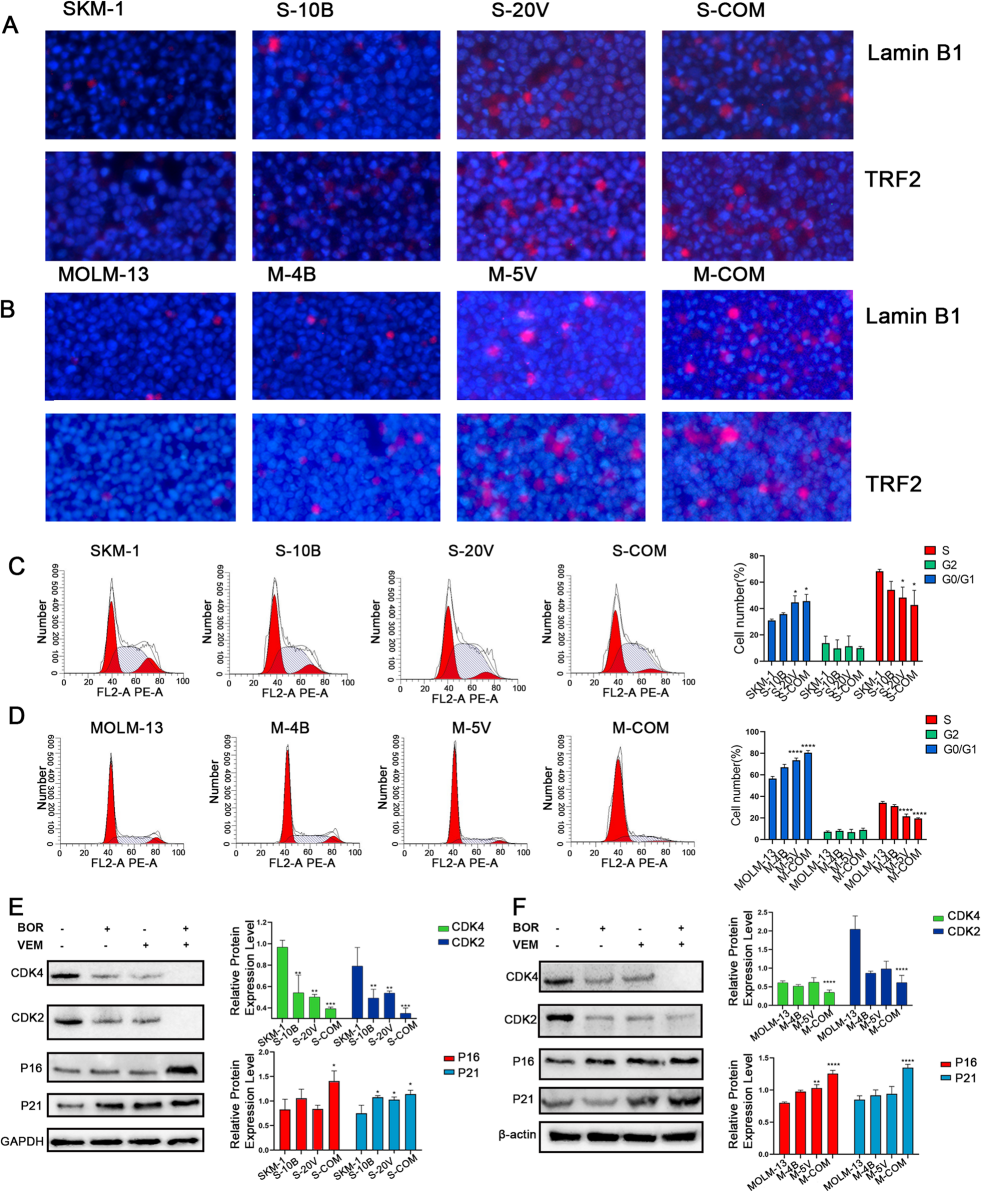
VEM in combination with BOR induce cellular senescence in AML. **A** LaminB1 and TRF2 in SKM-1 cells treated by VEM and/or BOR detected by IF; **B** LaminB1 and TRF2 in SKM-1 cells treated by VEM and/or BOR detected by IF; **C**–**D**, VEM in combination with BOR arrested the cell cycle at S phase in AML cells; **E**–**F** Expressions of cell cycle related protein (CDK2, CDK4, P16, and P21) were detected by western blot. GAPDH was used as a loading control

Lamin B1 and TRF2 were specifically observed in senescence cells. Our results indicated that VEM and/or BOR induced Lamin B1 and TRF2 in SKM-1 and MOLM-13 cells detected by Immunofluorescence (Fig. 4A–B). Flow cytometry was used to detect the cell cycle progress in each treatment group. Consequently, we found that VEM and/or BOR induced SKM-1 and MOLM-13 cell cycle arrest in G0/G1 phase (Fig. 4C–D). Meanwhile, Western blotting suggested that the combination of these drugs could promote AML cell cycle arrest at G1/G0 phase by promoting the expression of p16 and p21, and reducing the expression of CDK4 and CDK2 (Fig. 4E–F).

### Network pharmacology and RNA-seq analysis

**Fig. 5:**
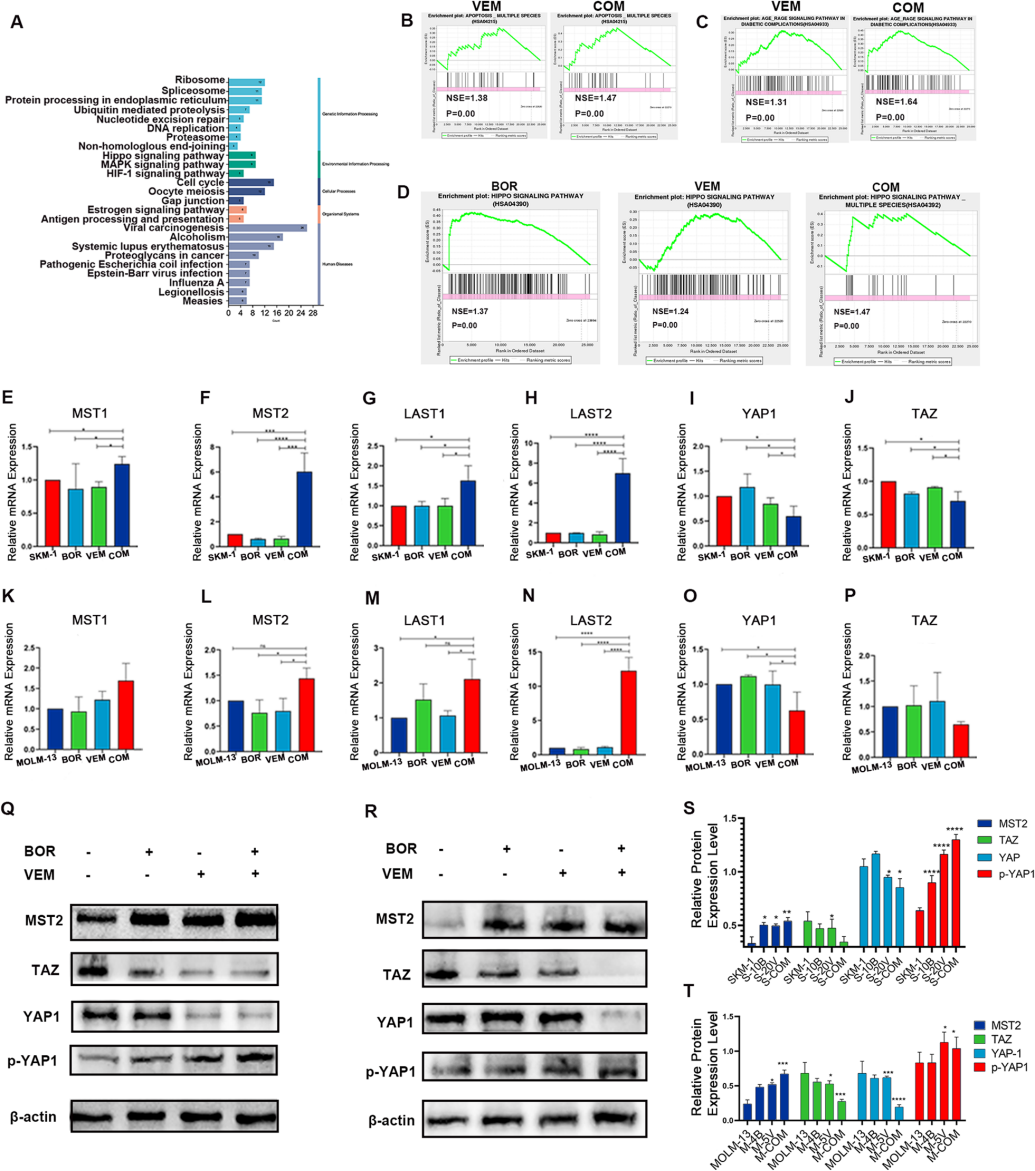
VEM and BOR treatment could turn on HIPPO signaling pathway in AML cell lines. **A** KEGG pathway enrichment analyses of the network pharmacology of VEM and BOR against AML-MDS; **B** Gene set enrichment analysis of apoptosis comparing SKM-1 cells treated VEM and drugs combination; **C** Gene set enrichment analysis of AGE-RAGE signaling pathway comparing SKM-1 cells treated VEM and drugs combination; **D** Gene set enrichment analysis of HIPPO signaling pathway comparing SKM-1 cells treated with BOR, VEM and drugs combination; **E**–**P** Relative expression level of genes in HIPPO signalin pathway (MST1/2, LAST1/2, YAP1, TAZ) was normalized to β-actin in AML cell lines by RT-qPCR; **Q**&**S**, Expressions of the genes in HIPPO signalin pathway (MST2, YAP1, TAZ) were detected in SKM-1 cells by western blot, β-actin was used as a loading control; **R**&**T** Expressions of the genes in HIPPO signalin pathway (MST2, YAP1, TAZ) were detected in MOLM-13 cells by western blot, β-actin was used as a loading control. *P < .05; **P < .01; ***P < .001; ****P < .001. Data was presented as mean ± SD of three independent experiments

To gain insights into the molecular mechanisms underlying BOR and VEM on AML cells, we first used the scientific and systematically strategy from network pharmacology. Using the BisoGenet plugin of Cytoscape3.7.1, the PPI networks of co-treatment drugs and AML disease targets were constructed respectively. The merged network of the above PPI network was constructed to further analyze the topological parameters by cytoNCA (Additional file 1: Fig. S3A). In the first screening, a hit-hub network was obtained using DC > 58 (Additional file1: Fig. S3B). The core PPI network with 144 nodes was eventually constructed by further screening with multiple topological parameters as described in the method (Additional file 1: Fig. S3C). Those144 core drugs-disease targets were analyzed by the Metascape platform to study GO and KEGG enrichment analyses (Fig. 5A) with the Hippo signaling pathway ranked first among all enriched pathways. In addition, we employed molecular docking, and we found that *BRAF* could bind to MST2 (Additional file 1: Fig. S4), that was consistent with previous literature [5].

Meanwhile, SKM-1 cells treated with VEM and BOR alone or in combination were collected for RNA-seq analysis. There were 181 up-regulated genes and 140 down-regulated genes in SKM-1 cells treated with VEM (Additional file 1: Fig. S3C and E). 447 up-regulated genes and 15 down-regulated genes were found in SKM-1 cells treated with BOR (Additional file 1: Fig. S3C and D). Differentially expressed genes (Additional file 1: Fig. S3C and F) were mainly enriched in apoptosis pathway (Fig. 5B, Additional file 1: Fig. S3I) and AGE-RAGE signaling pathway (Fig. 5C, Additional file 1: Fig. S3I) in AML cells treated with VEM and BOR. In the meantime, gene set enrichment analysis showed a significant enrichment of HIPPO signaling pathway (Fig. 5D) in cells treated by the combination of VEM and BOR.

Thus, network pharmacology and RNA-seq analysis suggested that VEM combined BOR might inhibit AML cells by turning on HIPPO signaling pathway.

### *Combination of VEM and BOR inhibited AML cells *via* HIPPO signaling pathway*

Given the indication from network pharmacology and RNA-seq analysis, we hypothesized that the combination of VEM and BOR could turn on HIPPO signaling pathway. The core HIPPO pathway comprises a kinase cascade, wherein an upstream kinase MST1/2 phosphorylates and activates a downstream kinase LAST1/2, resulting in the phosphorylation and subsequent inactivation of the transcriptional coactivator YAP/TAZ. Compared with VEM or BOR alone, the combination significantly upregulated the upstream genes (MST1/2, LAST1/2) of HIPPO signaling pathway, thereby inhibited the expression of YAP and TAZ, 2 key genes in HIPPO signaling pathway, at the mRNA level in both AML cell lines (Fig. 5E–P). This result was also verified by western blot assay. The combination significantly inhibited the expression of YAP1 and TAZ, while increased the MST2 expression and phosphorylation of YAP1 level in protein level (Fig. 5Q–T).

**Fig. 6:**
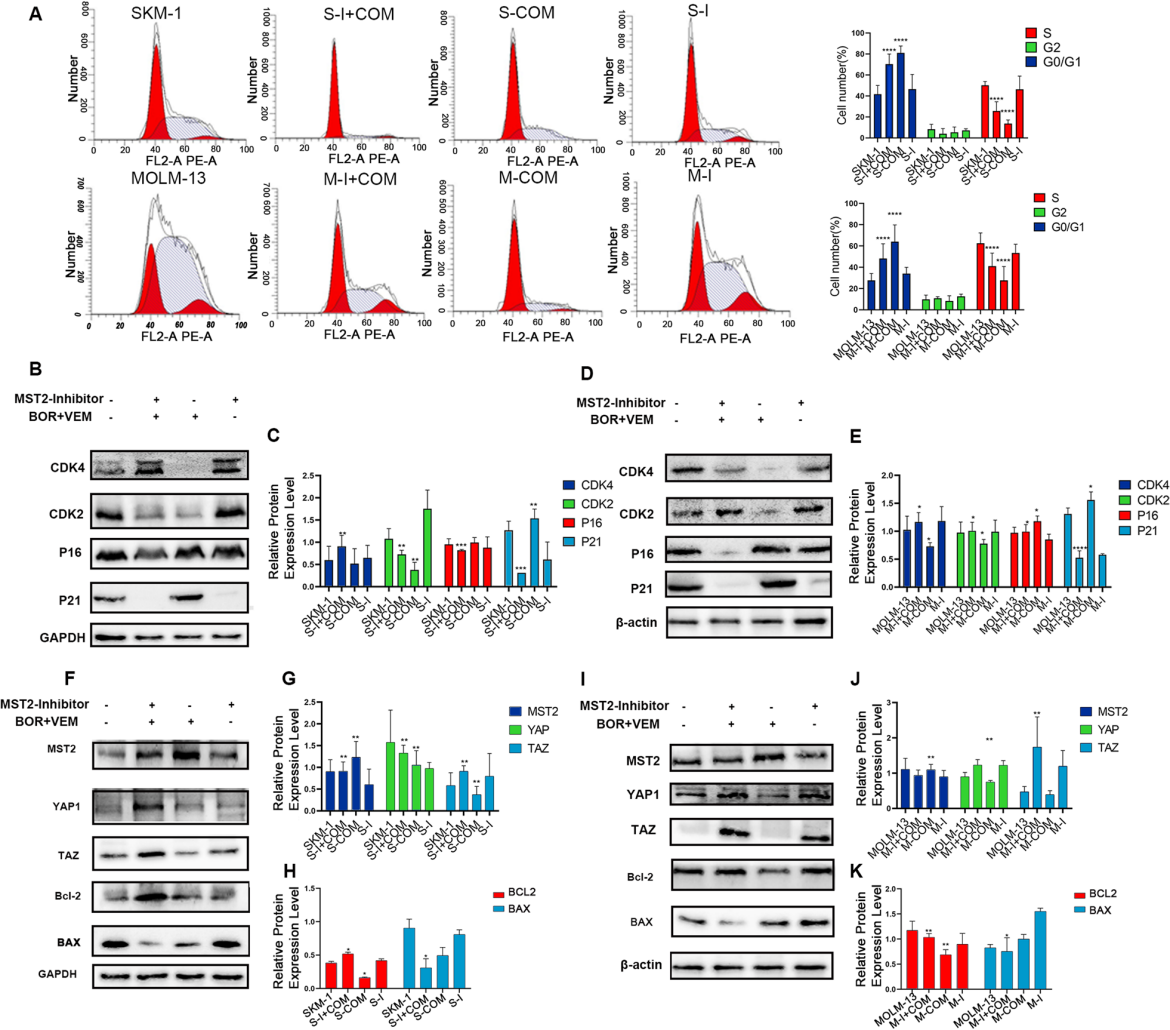
MST2 inhibitor reversed the anti-AML effects of VEM combined BOR. **A** MST2 inhibitor reversed the arrested of cell cycle at G0/G1 phase in AML cells; **B**–**C** Expressions of cell cycle related protein genes (CDK2, CDK4, P21,) were detected in SKM-1 cells by western blot, GAPDH was used as a loading control; **D**–**E** Expressions of cell cycle related protein genes (CDK2, CDK4, P21,) were detected in MOLM-13 cells by western blot, β-actin was used as a loading control; **F**–**H** Expressions of the genes in HIPPO signalin pathway (MST2, YAP1, TAZ) and apoptosis-related protein genes (BCL-2, BAX) were detected in SKM-1 cells by western blot, GAPDH was used as a loading control; **I**–**K** Expressions of the NEDD8, CUL5 and genes in HIPPO signalin pathway (MST2, YAP1, TAZ) and apoptosis-related protein genes (BCL-2, BAX) were detected in MOLM-13 cells by western blot, β-actin was used as a loading control. (COT-Control, COM-Drugs Combine, I-MST2 Inhibitor, I + COM-MST2 Inhibitor + Drugs Combine, *P < .05; **P < .01; ***P < .001; ****P < .001. Data was presented as mean ± SD of three independent experiments)

XMU-MP-1, a MST2 inhibitor, reversed the cell cycle stagnation in G0/G1 phase induced by combination treatment in both SKM-1 and MOLM-13 cells (Fig. 6A). In the meantime, cellular senescence related proteins p21 and p16 were down-regulated, while CDK4 and CDK2 were up-regulated (Fig. 6B–E), which suggested that cellular senescence was regulated by HIPPO signaling pathway.

Reversing assay also showed that XMU-MP-1 could inhibit the activation of HIPPO signaling pathway as demonstrated by the change of YAP1 and TAZ expression level (Fig. 6F–K). These observations suggest that VEM and BOR promoted the cellular senescence of sAML cell lines via HIPPO signaling pathway.

### *VEM combined with BOR induced senescence and inhibited growth of AML cells *in vivo

**Fig. 7:**
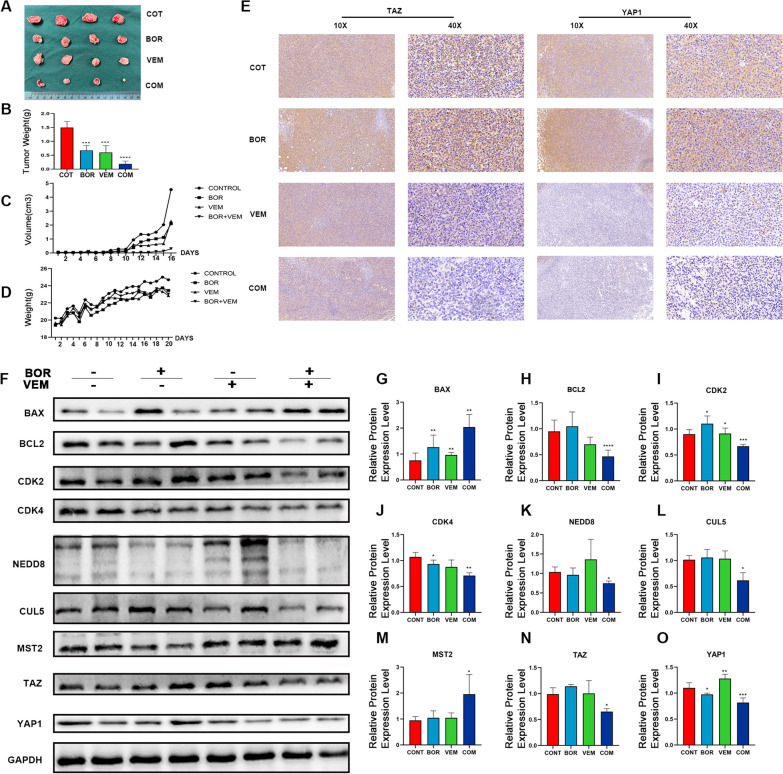
VEM synergistic BOR in anti-AML through HIPPO signaling pathway in vivo. After the administration, the tumors were excised, weighed, and photographed (**A**). The tumor weight (**B**), tumor volume (**C**) and weight of mice (**D**) were recorded every two days during the experiment, n = 4; **E**, the expression of YAP1 and TAZ was detected by immunohistochemistry in vivo; **F**–**O**, Expressions of the BAX, BCL-2, CDK2, CDK4, NEDD8, CUL5 and genes in HIPPO signaling pathway (MST2, YAP1, TAZ) were detected by western blot, GAPDH was used as a loading control. *P < .05; **P < .01; ***P < .001; ****P < .001. Data was presented as mean ± SD of three independent experiments

Xenograft mouse model was used to verify the anti-AML effect of VEM and BOR. As demonstrated in Fig. 7A, VEM and BOR single treatment significantly reduced the tumors size when compared with control group, and tumors volume were the smallest in combination group among all groups on day 21 (Fig. 7B and C). During the whole in vivo experiment, no significant change in body weight was noted among all groups, suggesting that VEM and BOR was well tolerated (Fig. 7D). In addition, HE staining confirmed that the combination of VEM and BOR could promote tumor cell necrosis without damaging the rest of other organs in mice (Additional file 1: Fig. S5).

RT-qPCR assay confirmed that the combination of BOR and VEM could enhance the mRNA expression of STK3 (MST2) (Additional file 1: Fig. S6G), and WB assay confirmed that the combination of BOR and VEM could enhance the expression of BAX and MST2, and reduce the expression of BCL-2, TAZ and YAP1 (Fig. 7F), which was consistent with the results in vitro. Immunohistochemical experiments also showed that the expression of TAZ and YAP1 was decreased in the combination group compared with BOR or VEM alone (Fig. 7E). Taken together, VEM combined with BOR showed a remarkable anti-AML effect in vivo and was well tolerated in mice model.

### Combing VEM and BOR could significantly reduce ROS generation in AML cells

Cancer cells have been shown to carry more reactive oxygen species (ROS) than normal tissue to support malignant behavior [17]. Thus, we also explored the effect of VEM combined with BOR on ROS generation in AML cells. We found that although VEM and BOR alone could reduce ROS generation in AML cells, the combination of BOR could significantly reduce ROS generation in AML cells (Additional file 1: Fig. S7).

## Discussion

Acute myeloid leukemia is characterized by the accumulation of bone marrow blasts [18], like many other cancers, is a disease commonly found in elderly people according to National Institutes of Health (NIH) Surveillance, Epidemiology and End Results (SEER) database [19]. Growing concern about the role of aging in leukemogenesis [20]. Aging is characterized by a progressive loss of physiological integrity, leading to impaired function and increased vulnerability to death. Researchers have identified some characteristics of aging, including epigenetic alterations, loss of proteostasis, mitochondrial dysfunction, and cellular senescence [21]. With the average human life expectancy increasing, the role of aging and senescence in the pathogenesis of AML has remained attention [22]. Meanwhile, due to the unknown pathogenesis of AML, the rapid progression of AML patients, poor chemotherapy effect and high recurrence rate, it is still an important topic to explore the pathogenic genes and the role of senescence of AML and new treatment options for AML.

Abnormal expression of *BRAF* gene has been found in multiple tumors, and its abnormal expression is associated with the pathogenesis of melanoma [23, 24] and thyroid cancer [25]. In 101 AML patients and 10 sAML patients, we found that *BRAF* was overexpressed in AML patients, and its high expression was associated with poor prognosis. At the same time, *BRAF* was decreased when AML patients achieved CR, but increased significantly when AML patients relapsed, which further suggested that *BRAF* may play a pathogenic role in AML.

In addition, age was correlated with *BRAF* expression, and older AML patients had higher *BRAF* expression levels, and older AML patients with high *BRAF* expression had the shortest OS. In the meantime, we found that high *BRAF* expression and TET2 and DNMT3A mutations were more commonly found in elderly AML patients, and that the survival time of these AML patients carrying TET2 and DNMT3A was shortened by high *BRAF* expression. Epigenetic regulators TET2 and DNMT3A benefit the accumulation cell damage to promote the tumorigenesis [26]. Therefore, we speculated that abnormal expression of *BRAF* might promote the development of sAML from the damaged aged MDS patients.

To explore the therapeutic potential of *BRAF* inhibitor VEM in AML cells, we proved that VEM could inhibit the proliferation of AML cells. Counting on previous research convinced that the combination of the BOR and the VEM as an efficient therapeutic approach for the treatment of thyroid cancer [27], and BOR was usually used to enhanced the efficiency in many therapies in AML [28, 29], we revealed the combination of VEM and BOR could synergistically inhibit AML cells.

Several researches had indicated that *BRAF* inhibitors were correlated to HIPPO signaling pathway. Lin et al. [30] unveiled the synthetic lethality of combined suppression of YAP1 (a key downstream effector of HIPPO pathway) and *BRAF* inhibitors as a promising strategy to enhance treatment response in human cancers with oncogenic activation of BRAF-MEK-ERK signaling pathway. In the meantime, Li et al. [24] revealed *BRAF* inhibitor VEM induced cell death via inhibiting the expression of YAP and downregulated HIPPO pathway. In the meantime, Network pharmacological analysis and RNA-seq analysis suggested that VEM and/or BOR simultaneously regulated HIPPO signaling pathway of AML cells.

Cellular senescence can be defined as a stable arrest of the cell cycle coupled to stereotyped phenotypic changes [31], senescence induction and senescent cells clearance has been considered as promising therapeutic strategy for cancer treatment. Multiple strategies have now been tested to safely and effectively induce cancer cell senescence based on known senescence inducers and effector pathways [32]. Researches revealed pro-apoptotic was one of strategies on the clearance senescent cells [33, 34].

Studies have shown that phosphorylation of YAP, key genes in HIPPO signaling pathway, can promote cell senescence. In ethanol-induced cellular senescence, up-regulation of YAP decreases senescence markers (P16) in hepatocytes. In human fibroblast cells, progressive loss of YAP promotes concomitant increase of senescence through P16 pathways in a TEAD-dependent manner; in context of cancer, the authors reported that in mesothelioma and liver cancer, YAP downregulation was also able to stimulate senescence [35]. Therefore, the transcriptional activity of its target gene was decreased in senescent cells when YAP/TAZ were phosphorylated and primarily located in the cytoplasm, its ability to self-repair and proliferate significantly decrease, its resistance to cellular senescence will also decrease, and apoptosis occurs [36].

Therefore, immunofluorescence and flow cytometry confirmed that *BRAF* inhibitor-VEM could induce cellular senescence by rescuing MST2 and p-YAP1 to turn on HIPPO signaling pathway, including upregulated the P16, P21, TRF2 and Lamin B1, promoting sAML cell lines arrested of cell cycle at G1/G0 phase, and enhanced by BOR. In addition, we used the proteasome inhibitor BOR to increase the intracellular expression level of MST2. Thus, our results suggested that VEM and/or BOR could synergistically induce senescence by inhibiting the proliferation of sAML cells, and promoting the apoptosis of sAML cells. Western blotting analysis convinced that the content of p-YAP1 in cytoplasm was increased in AML cell lines treated by VEM and/or BOR. Cellular senescence was induced with up-regulation of P21 and P16, companied with cell cycle related molecular CDK4 and CDK2 are downregulated.

Furthermore, considering the important role of mitochondrial where ROS comes from, proteins, lipids, and nucleic acids within cells are damaged by ROS, which are known to be harmful reactive particles. It usually appears in pathological processes when they are not scavenged on time [37]. We investigated the effect of VEM and BOR on ROS production in AML cells. We found that although BOR and VEM alone could reduce ROS in AML cells, the ROS was significantly reduced when both drugs were treated with AML cells.

Based on the results of in vitro experiments, we used Xenograft tumor model and treated these mice with VEM and/or BOR. We found that VEM could reduce the tumor burden in mice, and when VEM was combined with BOR, the tumor burden in mice was further reduced. In addition, mice treated with VEM and BOR had increased expression of MST2 and decreased expression of YAP and TAZ. Moreover, the expression of apoptosis-promoting factor BAX increased and the expression of apoptosis-antagonizing factor BCL-2 decreased. Therefore, we believe that *BRAF* inhibitors can cooperate with BOR to promote cellular senescence of sAML cells through turning on HIPPO signaling pathway, reducing ROS generation in sAML cells, thus inducing aging of sAML cells.

While there were certain limitations in this study, a retrospective design was employed to investigate the expression and prognostic value of *BRAF* in AML. Furthermore, it is imperative to conduct prospective and randomized clinical trials for further validation of the efficacy of VEM and BOR against AML.

## Conlusions

In summary, *BRAF* was characterized in high expression and negative clinical correlates in AML and MDS patients. And the *BRAF* inhibitor VEM could inhibit AML cells by inducing senescence through activating HIPPO signaling pathway. Analyzing the expression of *BRAF* may help predict prognosis and provide potential therapeutic target for AML and MDS patients.

### Supplementary Information

**Additional file 1:** Supplementary data.

## Data Availability

Data are available from the corresponding author upon reasonable request.

## Abbreviations

MDS: Myelodysplastic syndromes (MDS)
OS: Overall survival
AML: Acute myeloid leukemia
WBC: White blood cell count
PLT: Platelets count
Hb: Hemoglobin content
VEM: Vemurafenib
BOR: Bortezomib
BMMCs: Bone marrow mononuclear cells
HR-MDS: High risk MDS
LR-MDS: Low risk MDS
ELN: European LeulemieNet
HSCT: Hematopoietic stem cell transplantation
sAML: Secondary AML
CR: Complete remission
CI: Combination index
