# Supplementary material for: Vemurafenib induces senescence in acute myeloid leukemia and myelodysplastic syndrome by activating the HIPPO signaling pathway: implications for potential targeted therapy
Source: Biol Direct. 2024 Jan 4;19:6. doi: 10.1186/s13062-023-00451-0 (PMC10768477; doi:10.1186/s13062-023-00451-0)
Supplement: Supplementary file 1 — Additional file 1: Supplementary data. [file 13062_2023_451_MOESM1_ESM.docx]

**Supplementary materials and methods**

1.1 Network pharmacological analysis

Drug bank^[[1]](#endnote-1)^, SEA Search Server^[[2]](#endnote-2)^, Therapeutic Target Database^[[3]](#endnote-3)^, TargetNet^[[4]](#endnote-4)^, CANSAR^[[5]](#endnote-5)^, SwissTargetPrediction1^[[6]](#endnote-6)^ and PharmMapper2^[[7]](#endnote-7)^ were used to establish the targets of VEM and BOR. Genomic targets of MDS, AML and AML-MDS were obtained from DisGeNet^[[8]](#endnote-8)^, GeneCards3^[[9]](#endnote-9)^ and OMIM^[[10]](#endnote-10)^, and overlapping genes were collected. DAVID database5^[[11]](#endnote-11)^ was used to perform Gene Ontology (GO) and Kyoto Encyclopedia of Genes and Genomes (KEGG) pathway enrichment analyses.

1.2 Drugs

VEM was provided by Shanghai yuanye Bio-Technology Co.,Ltd . (Shanghai, China), was dissolved in dimethyl sulfoxide (DMSO, Sigma-Aldrich, St Louis, Missouri, USA) with a concentration of 10 mmol/L and stored at -80℃. BOR was provided by the First Affiliated Hospital of Chongqing Medical University, and stored at -80℃.

1.3 Cell viability assays

Cell viability was measured by Cell Counting kit-8 (CCK-8) assay (MCE, Shanghai, China). Briefly, cells were seeded at 5000 cells/100 µL and treated with different concentrations of VEM and BOR for 24, 48, and 72 h. CCK-8 reagent was added and incubated for 3 h. The absorbance at 450 nm was measured using a Multiskan Go Microplate Spectrophotometer (Thermo Fisher Scientific, United States). Cell proliferation inhibition rate was calculated based on the formula: absorbance of (control group - experimental group)/absorbance of (control group - blank group) × 100%.

1.4 Cell apoptosis analysis and cell cycle analysis

Since we measured the CI index of multiple drug concentration combinations on sAML cells, drug combination with a smaller CI index and a greater synergic effect were chosen in the following experiment. We applied BOR of 10nM concentration combined with VEM of 20uM to treat SKM-1 cells. MOLM-13 cells were treated with BOR at 4nM combined VEM at 5uM. For cell cycle analysis, cells were fixed with ice-cold 75% ethanol overnight at 4℃ and then incubated with 50 mg/ml of propidium iodide (PI) for 30 min at room temperature. For apoptosis, cells were incubated with 5 µl of Annexin V-FITC and 10 µl of PI, at 4◦C for 15 min in the dark. Cell cycle and apoptosis were analyzed using a flow cytometer (CytoFLEX, Beckman Coulter, United States).

1.5 ROS

The intracellular ROS levels were measured using a Reactive Oxygen Species Assay Kit (Beyotime Biotechnology, China); 2’, 7’-dichlorofluorescein-diacetate (DCFH-DA), which is easily oxidized to fluorescent dichlorofluorescein (DCF) by intracellular ROS, is its principal component, and therefore, the ROS levels were quantified. Briefly, the cells were seeded in 96-well plates as described above and exposed to VEM and/or BOR. Following the treatment, the cells were incubated with DCFH-DA for 20min at 37°C and then observed using fluorescence microscopy (Olympus) and measured at 488 nm excitation and 525 nm emission by a fluorescence spectrophotometer (BioTek).

1.6 RNA Isolation and Reverse Transcription-Quantitative PCR (RT -qPCR)

RNA extraction from cells using TRIzol reagent (Beyotime, China) according to the manufacturer’s instructions. cDNA synthesis using PrimeScript Reverse Transcription reagent kit (Takara, Japan). Quantitative PCR (qPCR) using a CFX96 TouchTM Real-Time PCR Detection System (Bio-Rad, Hercules, CA, United States). The following RT-qPCR parameters were used: 95◦C for 30 s; 95◦C for 5 s, and 60◦C for 30 s repeated over 40 cycles. All primers were synthesized by Tsingke (Beijing, China) and the sequences were shown in Supplementary table 1. Transcript levels were normalized toβ-actin expression and the target gene expression was calculated using the formula 2^-△△Ct^.

1.7 Western blot analysis and Antibodies.

Total protein from the cells was harvested using RIPA lysis buffer supplemented with 1 µM PMSF (Beyotime, Shanghai, China) and 30 µg protein was separated on a 10% SDS-polyacrylamide gradient gel. The proteins were transferred onto PVDF membranes and blocked with 5% non-fat milk in Tris Buffered Saline with Tween-20 (TBST) for 2 h at room temperature. The blots were then incubated with primary antibodies overnight at 4◦C. Membranes were then washed 3 times with TBST and incubated with secondary antibodies for 1 h at room temperature. Protein bands were visualized with an ECL kit (Advansta, United States) and the band intensity was analyzed using Vilber Fusion software (Fusion, FX5 Spectra, France). β-actin was used as a loading control. The following primary antibodies were used: rabbit anti-BCL-2 (YT0470) from ImmunoWay (Texas, United States). anti-Bcl-2-associated X protein (BAX; SC-20067) form Santa Cruz. Caspase-3 (bs-0081R) from Bioss (Beijing, China). CDK2 (H08211543) and CDK4 (H10082274) from Wanleibio (Shenyang, China). STK3 Antibody (T55622) and Phospho-YAP1 Antibody (T55743) from Abmart (Shanghai,China). YAP1 Rabbit pAb (A1002), TAZ Rabbit pAb (A8202), NEDD8 Rabbit pAb (A1163), and CUL5 Rabbit pAb (A5369) from Abclonal (Wuhan, China). Mouse anti-β-actin (KM9001) from Sungene (Tianjin, China). Horseradish peroxidase (HRP)-conjugated goat anti-rabbit IgG (A0239) and anti-mouse IgG (A0258) were purchased from Beyotime Biotechnology (Shanghai, China).

**Supplementary table 1 Primer sequence of genes.**

| GENE | Forward | Reverse |
| --- | --- | --- |
| BRAF | 5'-GAGTCTTCCTGCCCAACAA -3' | 5'-TGGTTTCTTCTCTCCATCCTG-3' |
| NEDD8 | 5'-ACAGTGGCAAGCAGATGAATGA-3' | 5'-TGAGCGACAGGGTAAAGAGGT-3' |
| CUL5 | 5'-TCAAGATGATACGGCTTTGCT-3' | 5'-GCTGCCCTGTTTACCCATTA-3' |
| STK4 | 5'-TCAGGAGTGCCAAAGGAGTG-3' | 5'-TGCCCATCTCATCACCCAC-3' |
| STK3 | 5'-GAATCCGACTTGGGGACCAT-3' | 5'-CGTTTTTGGACATAGGGAAGGG-3' |
| LAST1 | 5'-TGAAGAGGAGTGAAAAGCCAG-3' | 5'-TCAGCCTTAGCAGCATCAGA-3' |
| LAST2 | 5'-TGTGACTGGTGGAGTGTTGG-3' | 5'-TGGAATGTGGAGCGTGTTCT-3' |
| YAP | 5'-AGTTACCAACACTGGAGCAGG-3' | 5'-CAGGAAGTCATCTGGGGTTCG-3' |
| TAZ | 5'-GTCCTTCCTAACAGTCCGCC-3' | 5'-TTTCCGCATCTCCACAGCC-3' |
| CASPASE3 | 5'-TGCTGAAACAGTATGCCGACA-3' | 5'-CAAATTCTGTTGCCACCTTTCG-3' |
| BAX | 5'-AGGATCGAGCAGGGCGAATG-3' | 5'-TCAGCTTCTTGGTGGACGCA-3' |
| BCL2 | 5'-CTGCACCTGACGCCCTTC-3' | 5'-ACACATGACCCCACCGAAC-3' |
| P21 | 5'-CTGCCTTAGTCTCAGTTTGTGT-3' | 5'-AACCTCTCATTCAACCGCCTA-3' |
| β-actin | 5'-CATTGCCGACAGGATGCAG-3' | 5'-CGGAGTACTTGCGCTCAGGA-3' |

**Supplementary Figures**

**
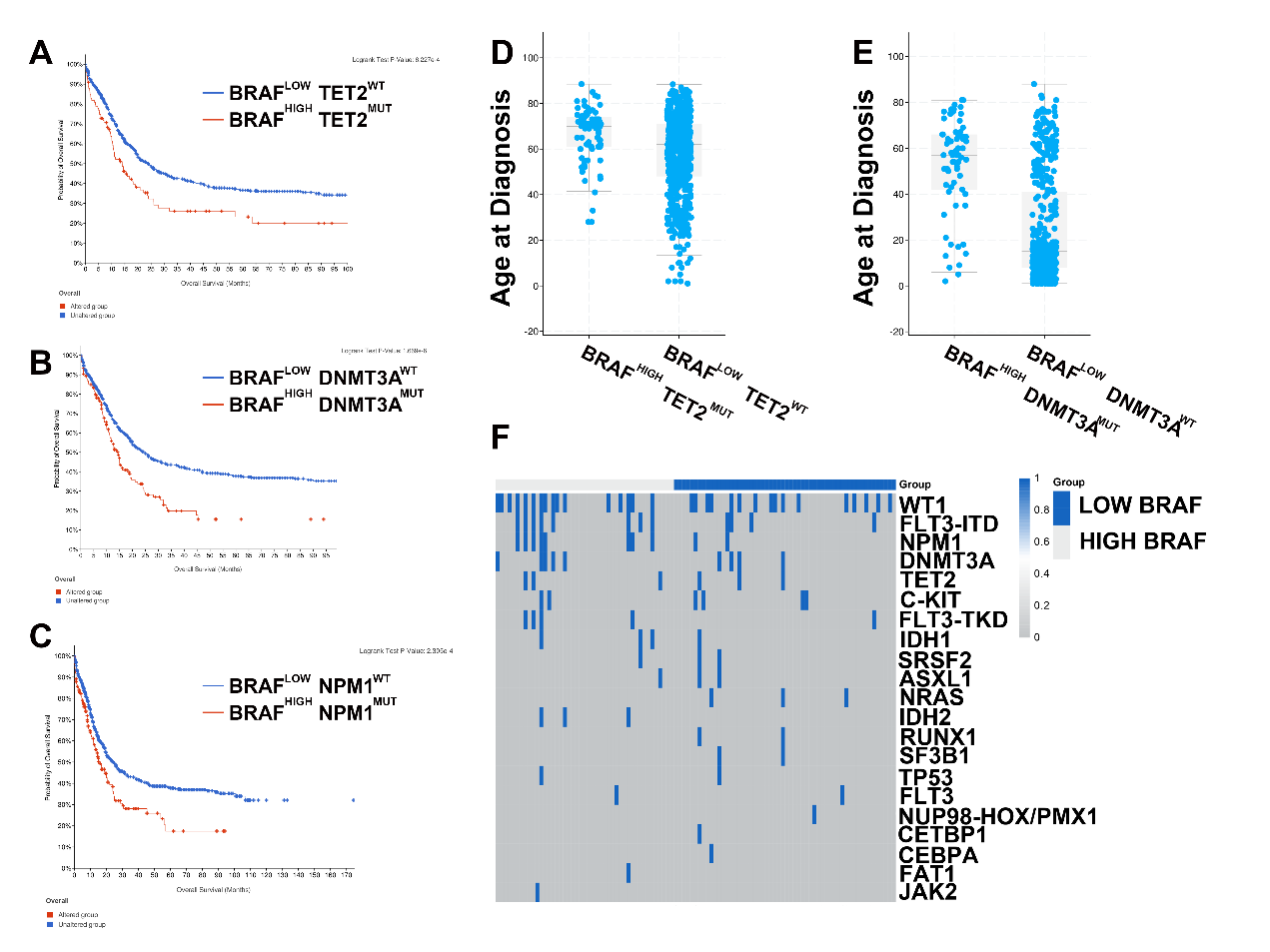
**

**Supplementary figure 1 (Figure S1) High expression of BRAF indicates poor prognosis in AML patients on TCGA database.** A, BRAF expression level on prognosis of AML patients with or without TET2 mutation in TCGA database; B, BRAF expression level on prognosis of AML patients with or without DNMT3A mutation in TCGA database; C, BRAF expression level on prognosis of AML patients with or without NPM1 mutation in TCGA database; D, Age at diagnosis of AML patients grouped by BRAF expression levels with or without TET2 mutation in TCGA database; E, Age at diagnosis of AML patients grouped by BRAF expression levels with or without DNMT3A mutation in TCGA database; F, Heatmap of AML patients grouped by BRAF expression level.


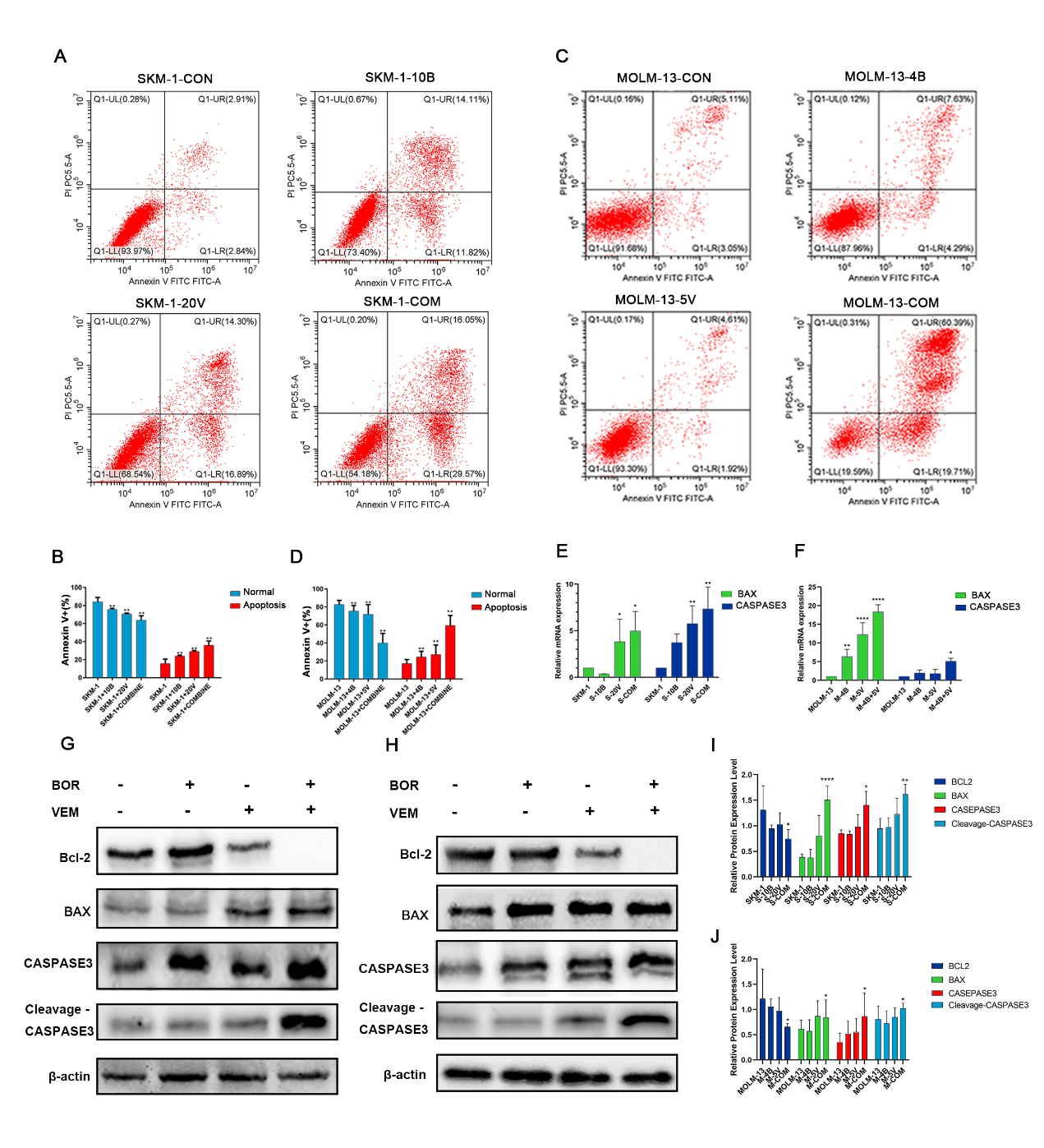


**Supplementary figure 2 (Figure S2) VEM in combination with BOR promote the apoptosis of AML cell lines.** A-B, VEM in combination with BOR can promote the apoptosis of SKM-1 cells; C-D, VEM in combination with BOR can promote the apoptosis of MOLM-13 cells; E, Relative expression level of BAX and CASPASE3 was normalized to β-actin in SKM-1 cells by RT-qPCR; F, Relative expression level of BAX and CASPASE3 was normalized to β-actin in MOLM-13 cells by RT-qPCR; G&I, Expressions of the apoptosis-related protein (BCL-2, BAX, CASEPASE3, Cleavage CASPASE3) were detected in SKM-1cells by western blot, β-actin was used as a loading control; H&J, Expressions of the apoptosis-related protein (BCL-2, BAX, CASEPASE3, Cleavage CASPASE3) were detected in MOLM-13 cells by western blot, β-actin was used as a loading control. (COT-Control, 10B-10 nM BOR, 20V-20 uM VEM, 4B-4 nM BOR, 5V-5 uM VEM, COM-Drugs Combine, *, P< .05; **, P< .01; ***, P< .001; ****, P< .001. Data was presented as mean ± SD of three independent experiments)


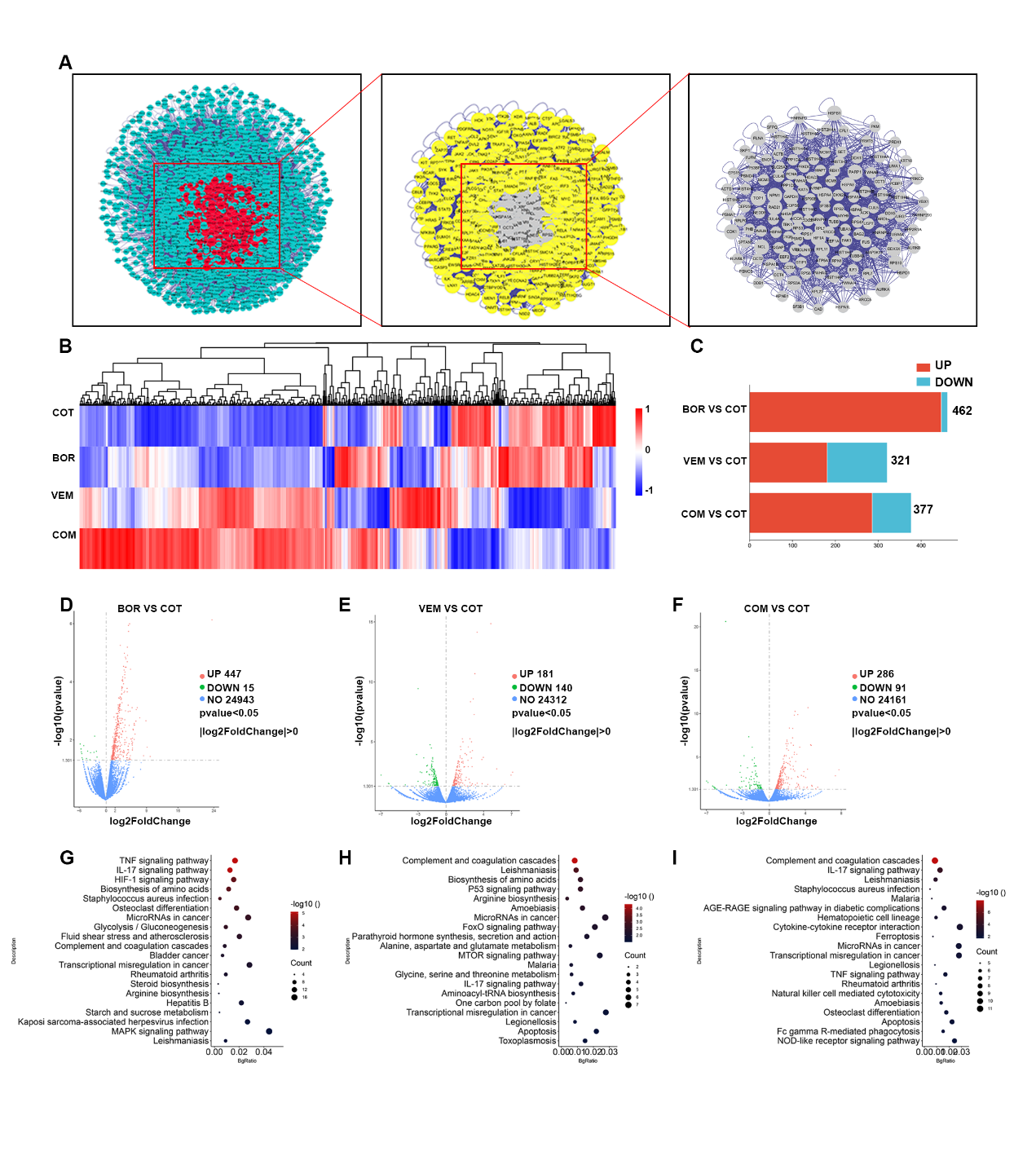


**Supplementary figure 3 (Figure S3)** **The mechanism of VEM combined with BOR inhibiting AML explored based on network pharmacology and RNA-seq analysis.** A, The PPI of drugs-disease merged target genes generated using BisoGenet; B, Heatmap of AML cells treated by VEM and BOR; C & D, Altered genes in AML cells treated with BOR; C & E, Altered genes in AML cells treated with VEM; C & F, Altered genes in AML cells treated with BOR and VEM; G, KEGG pathway enrichment analysis of upregulated genes in AML cells treated with BOR; H, KEGG pathway enrichment analysis of upregulated genes in AML cells treated with VEM; I, KEGG pathway enrichment analysis of upregulated genes in AML cells treated with BOR and VEM.


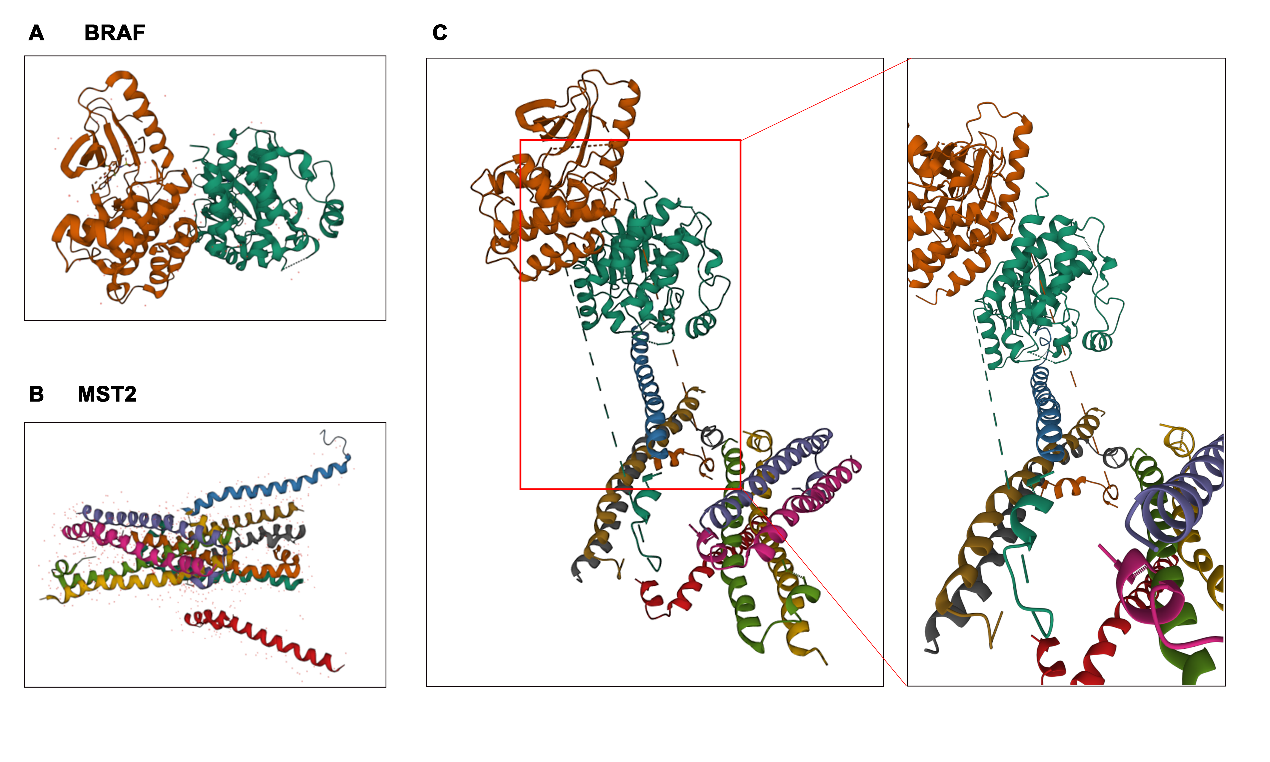


**Supplementary figure 4 (Figure S4) Schematic diagram of BRAF molecular docking with MST2.**

A, the molecular structure of BRAF; B, the molecular structure of MST2; C, the molecule of BRAF binds to the MST2.


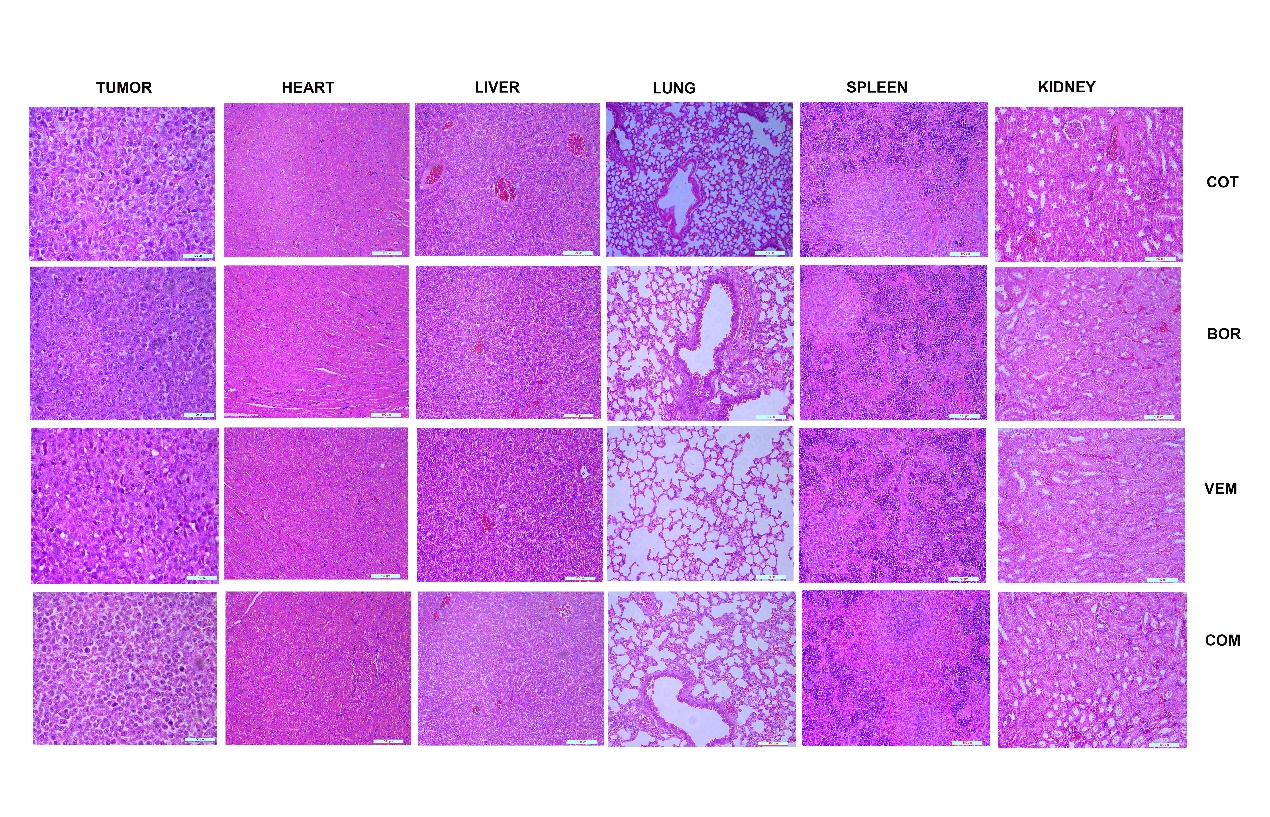


**Supplementary figure 5 (Figure S5) Histopathological detection of major organs in AML.1S xenograft model. Magnification, ×400.**


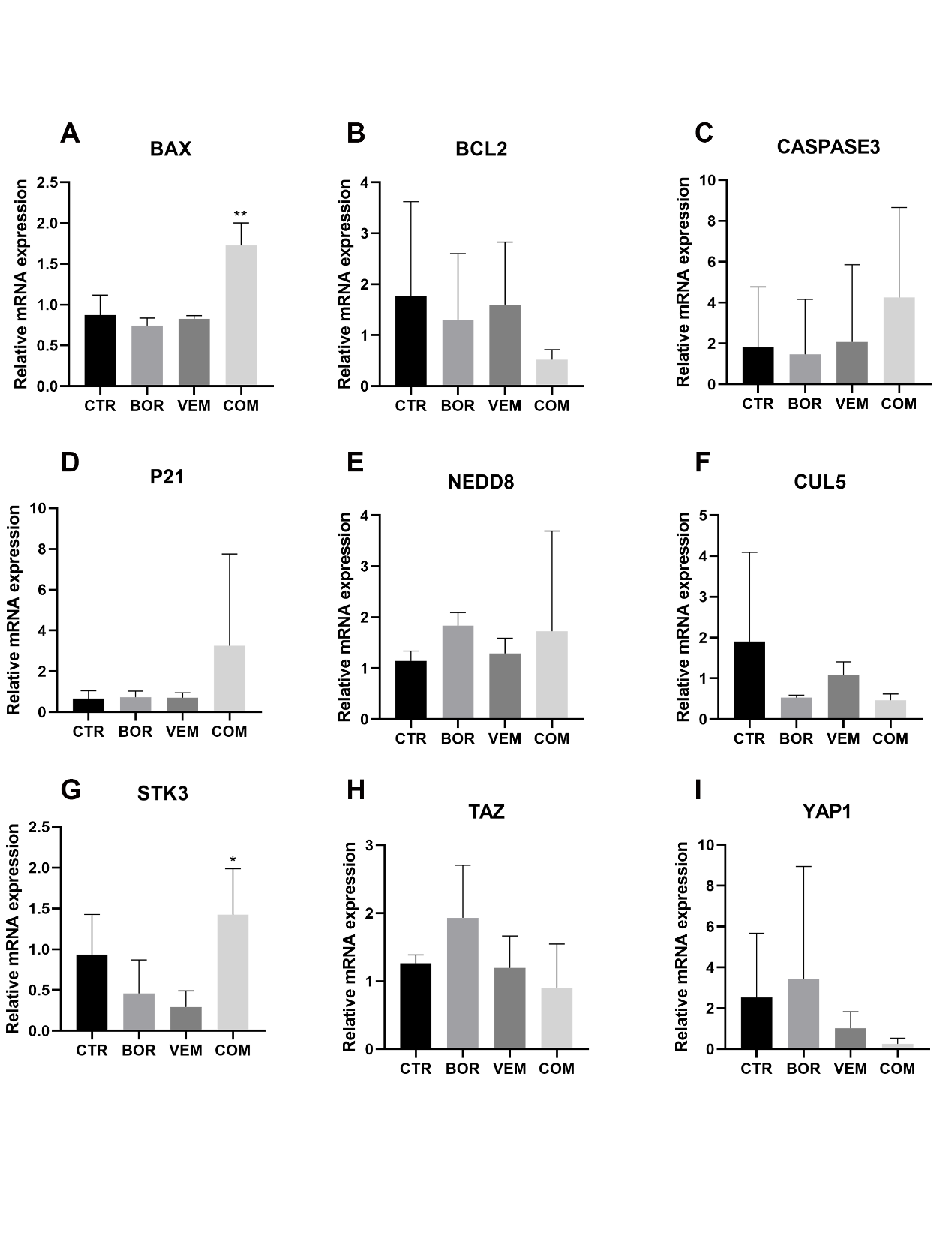


**Supplementary figure 6 (Figure S6) The gene mRNA expression in vivo.** A-I, Relative expression level of BAX, BCL-2, CASPASE3, P21, NEDD8, CUL5 and genes in HIPPO signaling pathway (MST2, YAP1, TAZ) was normalized to β-actin by RT-qPCR.


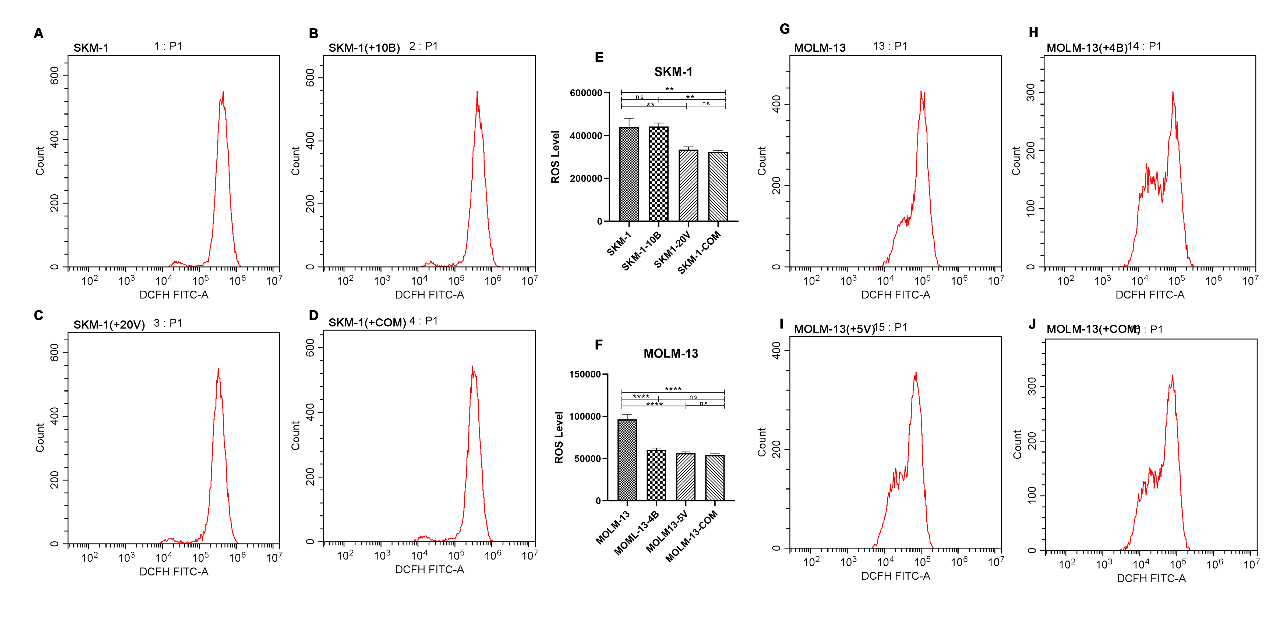


**Supplementary figure 7 (Figure S7) VEM and BOR decreased ROS in AML cell lines.** A-E, Intracellular ROS levels of SKM-1 cells treated with BOR and/or VEM were determined by flow cytometry; F-J, Intracellular ROS levels of MOLM-13 cells treated with BOR and/or VEM were determined by flow cytometry.

1. <https://go.drugbank.com/drugs/DB08881> [↑](#endnote-ref-1)
2. http://sea.bkslab.org/jobs/search_60467e5b-9c4c-44bb-9cf5-0d8633860a7f [↑](#endnote-ref-2)
3. http://db.idrblab.net/ttd/ [↑](#endnote-ref-3)
4. http://targetnet.scbdd.com/home/index/ [↑](#endnote-ref-4)
5. https://cansarblack.icr.ac.uk/#splash-about [↑](#endnote-ref-5)
6. http://swisstargetprediction.ch/ [↑](#endnote-ref-6)
7. http://www.lilab-ecust.cn/pharmmapper/check.html [↑](#endnote-ref-7)
8. https://www.disgenet.org/ [↑](#endnote-ref-8)
9. https://www.genecards.org/ [↑](#endnote-ref-9)
10. https://omim.org/ [↑](#endnote-ref-10)
11. [DAVID Functional Annotation Bioinformatics Microarray Analysis (ncifcrf.gov)](https://david.ncifcrf.gov/) [↑](#endnote-ref-11)
